# Supplementary material for: ACE-Neuro: A tailored exercise oncology program for neuro-oncology patients – Study protocol
Source: Contemp Clin Trials Commun. 2022 May 25;28:100925. doi: 10.1016/j.conctc.2022.100925 (PMC9198374; doi:10.1016/j.conctc.2022.100925)
Supplement: Multimedia component 1 [file mmc1.docx]

Supplementary File 1
Functional Fitness Assessment – In Person Assessment Overview

| Resting Vital Measures | Heart Rate Blood Pressure |
| --- | --- |
| Anthropometric Measures | Height *Measured using a Seca 217 Stadiometer* Weight *Measured using a 16 Health Carter Beam Scale* |
| Flexibility | Shoulder Flexion *Measured using a goniometer* Sit-and-Reach |
| Balance | Single Leg Stance |
| Muscular Strength | Grip Strength Measured using a hand grip dynamometer, following the Canadian Physical Activity, Fitness, and Lifestyle Approach (CPAFLA) |
| Muscular Endurance | 30-Second Sit-to-Stand |
| Cardiorespiratory Fitness | 2-Minute Step Test |
